# Report of Case of Acute Peritonitis, with Remarks

**Published:** 1851-07

**Authors:** Austin Flint


ART. V.—*Report of Case of Acute Peritonitis, with Remarks.* By AUSTIN FLINT, M. D. Read at the meeting of the Buffalo City Medical Association, June 17, 1851.

GENTLEMEN OF THE ASSOCIATION,

I will invite your attention to a brief report of a case of *acute peritonitis* which I have recently treated, in connection with which I will submit a few remarks on the interesting features of the case, and its management.

I was requested, on the evening of the 22d ultimo, to visit Mrs. H——, aged about 28, a woman of rather robust constitution, enjoying usually fine health, married some four years since, and having had two children, the youngest being 14 months old. Her last menstrual period had occurred two weeks after the proper period, and was protracted to eight days. The flowing was considerable, and accompanied with pain in the loins. She thought she might have aborted, although there had been no other evidences of pregnancy than the delay in the recurrence of the menstrual crisis. The flowing had terminated eight days prior to the present attack. In the mean time, she had felt some painful sensations, occasionally, in the loins, and lower part of abdomen. On the forenoon of the 22d, she was as well as usual, and had spent a portion of the time out of doors, in the garden. The weather was somewhat cold, and rainy. She had had also an increase of household duties for some time previously. She began to experience pain in the abdomen in the afternoon, which rapidly increased, soon becoming extremely severe, and accompanied by great prostration. I saw her at 5, P. M. When I entered the room, I supposed, from her aspect, that she was moribund. Her face was sunken, and had the pallor of death. The skin was cold, and bathed in perspiration. The pulse was almost inappreciable, *and not increased in frequency*. She complained of intense pain in the abdomen, extending to the inferior part of the chest. I was, at first, quite doubtful what to think of the case. The symptoms were not unlike, in appearance, those of the collapsed stage of epidemic cholera. I learned that she had had one dejection in the afternoon; not large, nor liquid. On exploration of the abdomen, I found exquisite universal tenderness; pain on pressure, manifestly not seated in the integument, increasing in a degree commensurate with the amount of pressure; moderate meteorism. I concluded it was a case of peritonitis, but I felt some hesitancy in the diagnosis, from the non-acceleration of the pulse, and from having not infrequently met with instances in which the local symptoms of peritoneal inflammation are simulated by a neuropathic affection of the integument, or subjacent structures. Guided by present indications, I administered stimulants freely, and a drachm of the tincture of opium. In an hour or two, the pulse was somewhat more developed, and the skin became a little warmer. The pain continued to be severe, being, in a greater or less degree, constant, but much increased in paroxysms with short intervals. In about three hours I directed an enema containing two drachms of the tincture of opium, and, shortly afterward, administered another drachm by the mouth. Little or no anodyne effect followed these measures. She passed the night without sleep, suffering acute pain. A sinapism was also applied over the abdomen. I left her late at night, but was summoned to visit her by day-break the next morning, and found the following symptoms:—Pulse well developed, and numbering 120. Abdomen moderately tympanitic. Respirations 30, and costal. Pain acute and constant, but increased in frequently returning paroxysms, causing her to cry out with the severity of suffering, although she is a woman of nerve. Tenderness extreme, and more marked in the right and left iliac regions, especially the former. She lay on her back with the limbs extended, but, by choice, with the head and shoulders much elevated. Thirst was urgent, and she constantly called for ice. I directed fomentation of hops to abdomen, and prescribed sulphate of morphia, gr. 1—4, to be repeated hourly, if the pain continued. She took a grain and a half in the space of seven hours. At ten A. M., Dr. Winne visited in consultation. It was agreed to apply a couple of leeches to the groins, and to continue the free use of morphia.

At evening she was somewhat more comfortable. She had experienced much difficulty in evacuating the bladder, owing to the pain occasioned by making the effort, but had succeeded after repeated trials in obtaining relief from that difficulty. Tongue was clean. Pulse 90. Skin warm. Considerable tympanites, and great tenderness. The severity of the pain, however, was lessened. It was agreed to apply a blister to the abdomen, and to continue the morphia in doses of gr. 1—2 every four or six hours.

24th. She passed a comfortable night. Slept several hours. Pain much diminished, and the severe paroxysms have ceased. Pulse 90. Skin warm and mellow. No dejection from the bowels since my first visit. Urinates with some difficuty, owing to the pain experienced by the effort. Aspect improved. Face less sunken.

Blister vesicated rather imperfectly. The morphia had been repeated in doses of gr. 1—2 every six hours.

It was agreed to continue the same plan of treatment, adding a warm cataplasm to be applied over the abdomen.

She passed a comfortable day. Pain at evening was not severe. Abdomen moderately tympanitic. Skin cool and dry. Pulse 90. No thirst. She has taken barley water for diet.

*Uterine flowing commenced in the forenoon of this day, and has continued through the day.* No dejection. Treatment continued.

25th. Passed a comfortable night. Suffered but little from pain. Uterine flowing continues. Abdomen moderately tympanitic. Tenderness diminished. Moves in bed with less pain. Urinates without difficulty. Aspect much improved. Skin mellow. Pulse 90. The morphia, in doses of gr. ss., every six hours, was steadily continued up to, and during this day. It occasioned a more sensibly anodyne effect than hitherto. She took some chicken broth with relish. No dejection.

26th. Passed a comfortable night. Abdominal distension and tenderness diminished. Uterine flowing lessened. It has been considerable. Pulse 90. Skin cool. No dejection save a very slight discharge of hard fecal matter passed without any painful effort. Some headach in the afternoon of this day. Morphia diminished to gr. 1—4, every six hours.

27th. In every respect comfortable except suffering from headach. Abdominal tenderness diminished. Pulse 85. No dejection having occurred, at noon an enema of warm water was given, which was retained; and the same measure was repeated at evening, and then followed by a tolerably large fecal evacuation, containing scybala, and voided without pain. The morphia was omitted. Cataplasms, which have been steadily continued, were still directed. The headach was relieved after the evacuation.

28th. Found her extremely comfortable. Tenderness much diminished. No pain. Pulse normal. Appetite improved. Flowing has ceased. No remedies were prescribed, but the cataplasms continued.

On the 59th, found no untoward symptoms, and discontinued attendance on this day. Dr. Winne continued to attend in consultation in the case until the severity of the symptoms ceased.

Up to the present time, June 17th, the patient has not required farther medicinal measures. She has continued to gain strength, and her usual healthy aspect is nearly restored. Soreness of the abdomen, on exercise, has continued, but progressively diminishing. The bowels have acted regularly. She has been careful to avoid undue exercise, and, at the present time, lies down a portion of the day.

The history of this case suggests various inquiries and reflections. I have not, however, leisure to indulge many remarks, nor do I wish to run the risk of occupying too long the attention of the Association.

The doubt may possibly be entertained whether the disease was acute peritonitis. I am well aware that a neuropathic condition, to which I have already alluded, may, without due knowledge and experience, be mistaken for an inflammation of the peritoneum. Such cases are as common as idiopathic peritonitis is rare. It was sufficiently clear to my own mind, and, I may add, Dr. Winne, who attended in consultation, was equally satisfied, that the affection was not of that description, but involved acute inflammation; and, indeed, the early symptoms appeared to denote so much gravity of disease, that a speedily fatal issue would not have taken us by surprise. Some of the symptoms, however, which usually attend acute peritonitis were absent. The attack was not ushered in by a chill. Cough was not present. Vomiting occurred but once, slightly, on the second day; the pulse was not so much increased in frequency as I have noticed in other cases that have fallen under my observation.

The great prostration, and the appearances of impending dissolution, although not peculiar to the sudden development of acute peritonitis in this particular instance, form an interesting feature of the case. We can understand why the system should give evidences of so severe, and almost overwhelming shock, when we consider the great extent of peritoneal surface and that the inflammatory attack was probably universal, or, at least, extending over a large space. Considered in this view, the affection is not unlike an extensive burn, or scald, which, as we know, is attended with extreme depression of the vital forces, death sometimes taking place without reaction having occurred. An amount of external surface inflamed equal to that of the peritoneum involved in this instance, would, perhaps, have been characterized by analogous symptoms.

The origin of the disease appeared to involve a morbid condition of the uterus and its appendages. The attack was preceded by menstrual disorder, and possibly the loss of an ovum in the early stage of uterine life. The tenderness was most marked over the ovaries. This morbid condition induced the predisposition, and the exposure to cold and wet, probably, was the exciting cause.

But the treatment of the case is the most important point connected with the history. It will be recollected by the members of the Association, that some years ago, Dr. Graves, of Dublin, reported several cases of peritonitis, from intestinal perforation, which were treated with large doses of opium, In one case, if I recollect rightly, the treatment was successful, and in the other cases the effect was such as to encourage the farther trial of the plan adopted. The object in this mode of practice was to arrest entirely the peristaltic movements of the intestines, and thus to favor adhesions of the peritoneal surfaces surrounding the perforation. I have myself witnessed the advantage of this method in a case of perforation, which I attended, some years ago, in consultation with Dr. White, of this city, in which, by continued use of large doses of opiates the bowels were kept quiescent for seven or eight days, and, in the mean time, the symptoms, which at first were almost as grave as possible, afforded strong encouragement of recovery. Yielding to importunities to move the bowels, the friends being outraged at the course pursued, a fresh development of inflammation ensued, probably from rupture of the adhesions, and a re-establishment of the perforation, and the disease speedily proved fatal. In reflecting on these cases, and the practical views therewith associated, it has seemed to me that the same therapeutical principles are measurably applicable to acute peritonitis from other causes than perforation. In the treatment of all inflammations the first object, and certainly one as important as any, is to keep the inflamed parts as quiescent as possible. We can appreciate the importance of this object when the inflamed parts are open to inspection; and analogy should teach us that it is not less desirable when the tissues inflamed are concealed from view. This object is recognized in wounds of the intestine made by operations, or accidents, and it is inculcated by the best surgical authorities.

In accordance with these ideas, I have for some time resolved to treat the first case of acute peritonitis that came under my charge by large doses of opium, or morphia, abstaining from cathartics, and adopting such other measures as the symptoms might appear to indicate, applying the same principles that have been recommended in cases of perforation, and in wounds of the abdomen, and for precisely the same end, viz.—to keep the inflamed parts in as perfect a state of quietude as practicable. This plan was pursued in the case the history of which has been given, the gentleman associated as counseling physician, entertaining similar opinions respecting its propriety. The bowels were kept quiescent by morphia for *five* days, no evacuation occurring during this period except one very slight discharge of solid fecal matter. At the end of this time, they were moved by an enema, the symptoms denoting acute inflammation having for the most part subsided. Aside from the laudanum and morphia, it will be observed that the whole treatment consisted of two enemas, two leeches, a blister which vesicated imperfectly, and hot cataplasms. The inflammatory action probably subsided and disappeared by resolution, without much effusion of fibrin and serum. Certainly, whether we take into view the duration of the disease, the rapidly progressive abatement of the symptoms, or the speedy convalescence, which has not been marked by any untoward circumstance, and has not required a grain of medicine, the career of the disease presents as fine an exhibition of the restorative energies of nature as could well be conceived of. For my own part, I have seldom, if ever, witnessed the progress and issue of a severe affection with greater satisfaction.

A word or two, in conclusion, respecting bloodletting. The condition of the pulse, etc., in this case appeared to forbid venæsection, notwithstanding the patient had a pretty robust constitution, and was attacked in good health. Local depletion, moreover, was very sparingly employed, two leeches only being applied. In connection with this subject it is an interesting fact that uterine flowing took place on the third day after the attack, and continued up to convalescence. The reflection will naturally arise, that this hæmorrhage may have exerted an important agency in the recovery; and the inference appears to my mind not irrational, that the occcurrence of this spontaneous depletion affords an argument in behalf of the propriety of local bloodletting, under similar circumstances, when the offices of nature in that way are withheld.

